# Supplementary material for: Intrapatient Comparison of Coblation versus Electrocautery Tonsillectomy in Children: A Randomized, Controlled Trial
Source: J Clin Med. 2022 Aug 4;11(15):4561. doi: 10.3390/jcm11154561 (PMC9369690; doi:10.3390/jcm11154561)
Supplement: Supplementary file 1 [file jcm-11-04561-s001.zip › jcm-1770810-supplementary.pdf]

Supplementary Table S1. Multivariate analysis of operation time on coblation and electrocautery sides.

Characteristic	Coblation side, seconds	*p*-value	Electrocautery side, seconds	*p*-value
Total	113.4 ± 50.1		268.9 ± 112.5	
Sex		0.075		0.019
Female	110.1 ± 9.6		235.0 ± 16.3	
Male	115.1± 9.2		285.9 ± 22.1	
Age, years		0.765		0.318
6-7	112.8 ± 11.9		233.5 ± 51.7	
8-10	103.0 ± 7.4		272.5 ± 20.2	
11-17	127.4 ± 6.5		317.6 ± 17.8	
Indication of surgery		0.904		0.046
Sleep-disordered breathing	115.5 ± 9.4		260.4 ± 20.1	
Recurrent tonsillitis	100.3 ± 7.5		324.3 ± 23.1	
Allergic rhinitis		0.181		0.838
No	115.2 ± 11.2		253.7 ± 25.0	
Yes	112.6 ± 8.3		276.6 ± 18.5	
KOSA-18 score		0.638		0.449
< 60	114.0 ± 7.9		276.1 ± 18.9	
60 – 80	123.2 ± 14.2		245.5 ± 27.6	
≥ 80	78.0 ± 2.6		260.0 ± 27.6	
Tonsil size		0.176		0.496
Grade 2	109.5 ± 6.6		267.5 ± 24.9	
Grade 3	110.9 ± 5.3		283.8 ± 19.4	
Grade 4	115.5 ± 11.4		259.5 ± 22.0	
Tonsillar adhesion		< 0.001		< 0.001
Mild	71.4 ± 2.4		169.7 ± 15.3	
Moderate	109.7 ± 5.0		288.1 ±16.1	
Severe	185.8 ± 9.5		370.0 ±17.5	

The values are presented as mean ± standard error.

Abbreviations: KOSA-18— Korean version of the Obstructive Sleep Apnea-18 questionnaire.

Supplementary Table S2. Daily pain scores, visual analog scale, on the coblation and electrocautery tonsillectomy sides in patients with sleep-disordered breathing (n = 26).

Postoperative day	Coblation side, VAS	Electrocautery side, VAS	*p*-value
0	4.0 ± 0.5	6.4 ± 0.5	< 0.001
1	4.1 ± 0.5	5.3 ± 0.6	0.064
2	4.5 ± 0.5	5.8 ± 0.6	0.022
3	3.9 ± 0.5	5.2 ± 0.5	0.006
4	3.2 ± 0.5	4.6 ± 0.5	0.007
5	3.3 ± 0.5	4.8 ± 0.5	0.004
6	3.4 ± 0.5	3.6 ± 0.4	0.647
7	2.8 ± 0.5	3.9 ± 0.5	0.060
8	2.4 ± 0.4	3.1 ± 0.5	0.065
9	2.2 ± 0.4	2.6 ± 0.5	0.036
10	1.5 ± 0.4	2.0 ± 0.4	0.091

The values are presented as mean ± standard error.

Abbreviations: VAS— Visual Analogue Scale.
